# Supplementary material for: DNA and RNA Alterations Associated with Colorectal Peritoneal Metastases: A Systematic Review
Source: Cancers (Basel). 2023 Jan 16;15(2):549. doi: 10.3390/cancers15020549 (PMC9856984; doi:10.3390/cancers15020549)
Supplement: Supplementary file 1 [file cancers-15-00549-s001.zip › cancers-2124667-supplementary.pdf]

Supplementary Table S1. Overview investigated genes with conclusions.

Name gene	Specification of Genetic Alteration Location	References	Conclusions Formulated by the Authors	Significantly Associated with PM?	Overall Conclusion Formulated by the Reviewers Based on Included Studies
Androgen receptor (AR)	Not specified	Lee et al. [30]	*AR* mutation was detected more frequently in patients with PM.	Yes	*AR* mutation can be associated with PM, but there is not enough evidence to formulate this conclusion. More research is necessary.
ASXL Transcriptional Regulator 1 (*ASXL1*)	Not specified	Lee et al. [30]	*ASXL1* mutation was detected more frequently in patients with PM.	Yes	*ASXL1* mutation can be associated with PM, but there is not enough evidence to formulate this conclusion. More research is necessary.
AT-Rich Interaction Domain 1A (*ARID1A*)	Not specified	Lan et al. [29]	No conclusion formulated.	N/A	*ARID1A* mutation can be associated with PM, but there is not enough evidence to formulate this conclusion. More research is necessary.
		Lee et al. [30]	ARID1A mutation was detected more frequently in patients with PM.	Yes	
Proto-oncogene B-Raf (*BRAF*)	Not specified	Atreya et al. [43]	*BRAF* mutant tumors have more common PM, not significant.	No	*BRAF* mutant tumors might be more likely to have PM, and mutations in *BRAF* might be higher for patients with PM compared to without.
		Lan et al. [29]	No conclusion formulated.	N/A	
		Prasanna et al. [45]	*BRAF* mutant CRC showed higher incidence of PM.	Yes	
		Roberto et al. [46]	*BRAF* mutant right-sided CRC was more likely to occur with PM.	Yes	
		Tran et al. [48]	*BRAF* mutant tumors had higher rates of PM.	Yes	
	Codon 600	Christensen et al. [44]	*BRAF* mutations were not associated with the presence of PM.	No	
		Kawazoe et al. [27]	*BRAF* mutated tumors were more likely to develop PM.	Yes	

Name gene	Specification of Genetic Alteration Location	References	Conclusions Formulated by the Authors	Significantly Associated with PM?	Overall Conclusion Formulated by the Reviewers Based on Included Studies
		Sasaki et al. [33]	The PM group had a higher incidence of *BRAF* mutations.	Yes	
		Shelygin et al. [35]	No differences were observed between PM and no PM group.	No	
		Taniguchi et al. [39]	Mutations in *BRAF* were higher for patients with PM.	Yes	
		Yokota et al. [40]	*BRAF*-mutated tumors disseminate more often to the peritoneum.	Yes	
	Codon 600, exon 11 and 15	Bruzzi et al. [21]	Trend for a higher rate of PM in *BRAF* mutant patients, not significant.	No	
	Codon 600, exon 15	Cheng et al. [22]	Patients with *BRAF*V600E mutation had a higher frequency of PM.	Yes	
		Sayagués et al. [34]	*BRAF* mutated tumors were associated with PM.	Yes	
		Yaeger et al. [49]	PM was more common in *BRAF* mutant cases.	Yes	
	Codon 600 and 594	Smith et al. [37]	A significant association between *BRAF* status and PM was found, although this association did not withstand correction for multiple testing.	No	
	All exons	He et al. [23]	*BRAF* mutated tumors were more likely to develop PM (trend), not significant.	No	
Kinesin Family Member 18A (*Kif18A*)	Not specified	Nagahara et al. [31]	*Kif18A* overexpression in CRC correlates with PM.	Yes	*Kif18A* overexpression can be associated with PM, but there is not enough evidence to formulate this conclusion. More research is necessary
RAS KRAS/NRAS	Not specified	Christensen et al. [44]	No association.	No	

Name gene	Specification of Genetic Alteration Location	References	Conclusions Formulated by the Authors	Significantly Associated with PM?	Overall Conclusion Formulated by the Reviewers Based on Included Studies
		Lan et al. [28]	The proportion of PM was higher in patients whose tumors carried a RAS pathway mutation. Tumors with a *KRAS* mutation had a trend toward a higher proportion of PM.	Yes	There is not enough evidence that *KRAS*/*NRAS* mutant tumors are associated with a higher rate of PM. PM is not more seen in RAS pathway-mutated tumors.
		Lan et al. [29]	No conclusion formulated.	N/A	
	Exon 2, 3 and 4	Bruzzi et al. [21]	No higher rate of PM in RAS mutant tumors.	No	
		Sayagués et al. [34]	No association between *KRAS*/*NRAS* mutation status and PM.	No	
	KRAS codon 61,146 + *NRAS* codon 12,13 and 61	Kawazoe et al. [27]	No differences for PM according to RAS mutation.	No	
	KRAS codon 12,13,61 + *NRAS* codon 12, 61	Smith et al. [37]	No association between *KRAS*/*NRAS* mutation status and PM.	No	
KRAS	*Not specified*	Christensen et al. [44]	No association.	No	
		Lan et al. [28]	The proportion of PM was higher in patients whose tumors carried a RAS pathway mutation. Tumors with a *KRAS* mutation had a trend toward a higher proportion of PM.	Yes	
		Lan et al. [29]	No conclusion formulated.	N/A	
	Codon 12, 13	Sasaki et al. [33]	No association between *KRAS* mutation status and PM.	No	
	Codon 12, 13 and 61	Yokota et al. [40]	No association.	No	

Name gene	Specification of Genetic Alteration Location	References	Conclusions Formulated by the Authors	Significantly Associated with PM?	Overall Conclusion Formulated by the Reviewers Based on Included Studies
	Codon 12/13 exon 2, Codon 59/61 exon 3, Codon 117/146 exon 4	Zihui Yong et al. [51]	PM was associated with *KRAS* mutant tumors.	Yes	
	All exons	He t al. [23]	Mutant *KRAS* tumors have a relevance with PM. *KRAS* codon 12 mutation is associated with PM and patients with PM tent to carry a mutant *KRAS* G12D.	Yes	
NIMA Related Kinase 2 (*NEK2*)	Not specified	Takahashi et al. [38]	High *NEK2* expression shows greater PM.	Yes	*NEK2* expression can be associated with PM, but there is not enough evidence to formulate this conclusion. More research is necessary.
MET Transcriptional Regulator MACC1 (*MACC1*)	Not specified	Shirahata et al. [42]	High *MACC1* expression shows correlation with PM.	Yes	*MACC1* expression can be associated with PM, but there is not enough evidence to formulate this conclusion. More research is necessary.
Paired Box 5 (*PAX5*)	Not specified	Lee et al. [30]	*PAX5* mutation was detected more frequently in patients with PM.	Yes	*PAX5* mutation can be associated with PM, but there is not enough evidence to formulate this conclusion. More research is necessary.
phosphatidylinositol-4,5-bisphosphate 3-kinase catalytic	Not specified	Christensen et al. [44]	*PIK3CA* mutation was associated with absence of PM and a decreased hazard of developing PM.	No	*PIK3CA* mutation is not associated with PM, and the presence of a mutation is

Name gene	Specification of Genetic Alteration Location	References	Conclusions Formulated by the Authors	Significantly Associated with PM?	Overall Conclusion Formulated by the Reviewers Based on Included Studies
subunit alpha (*PIK3CA)*		Lan et al. [28]	No association between PM and presence of PI3K pathway mutation.	No	possibly associated with a decreased change of developing PM.
		Lan et al. [29]	No conclusion formulated.	N/A	
		Smith et al. [37]	No differences were observed between PM and no PM group.	No	
	Exon 9 and 20	Sasaki et al. [33]	No differences were observed between PM and no PM group.	No	
	Codon 542, 545, 1047	Shelygin et al. [35]	No differences were observed between PM and no PM group.	No	
	Codon 542, 546, 1047	Taniguchi et al. [39]	No conclusion formulated.	N/A	
PKHD1 Ciliary IPT Domain Containing Fibrocystin/Polyductin (*PKHD1*)	Not specified	Lee et al. [30]	*PKHD1* mutation was detected more frequently in patients with PM.	Yes	*PKHD1* mutation can be associated with PM, but there is not enough evidence to formulate this conclusion. More research is necessary.
Regenerating Family Member 1 Alpha (*REG1A*)	Not specified	Astrosini et al. [20]	*REG1A* expression levels highly correlated with formation of PM.	Yes	*REG1A* expression can be associated with PM, but there is not enough evidence to formulate this conclusion. More research is necessary.
Ret Proto-Oncogene (*RET*)	Not specified	Yang et al. [50]	The presence of *RET* mutations was associated with PM.	Yes	RET mutation can be associated with PM, but there is not enough evidence to formulate this conclusion. More research is necessary.
Histone acetyltransferase (*Tip60*)	Not specified	Sakuraba et al. [32]	Downregulation of *Tip60* shows correlation with PM.	Yes	*Tip60* downregulation can be associated with PM, but there is not enough evidence

Name gene	Specification of Genetic Alteration Location	References	Conclusions Formulated by the Authors	Significantly Associated with PM?	Overall Conclusion Formulated by the Reviewers Based on Included Studies
					to formulate this conclusion. More research is necessary.
Tumor protein P53 (*TP53*)	Not specified	Lan et al. [29]	For patients with PM, the frequency of mutations was the highest in *TP53*.	No	*TP53* mutations are possibly detected more frequently in patients with PM, but further research is necessary to identify the association
		Lee et al. [30]	*TP53* mutation was detected more frequently in patients with PM.	Yes	
		Sayagués et al. [34]	No associations were found between *TP53* mutation and PM.	No	
		Sjo et al. [36]	PM was associated with mutations in *TP53.*	Yes	
Ubiquitin Protein Ligase E3 Component N-Recognin 5 (*UBR5*)	Not specified	Lee et al. [30]	*UBR5* mutation was detected more frequently in patients with PM.	Yes	*UBR5* mutation can be associated with PM, but there is not enough evidence to formulate this conclusion. More research is necessary.
Vimentin	Not specified	Shirahata et al. [41]	There is a trend toward developing PM and vimentin methylation, not significant.	No	Vimentin methylation can be possibly associated with PM, but further research is necessary.

CRC, colorectal cancer; PM, peritoneal metastases.
